# Exercise Training Increases Resting Calf Muscle Oxygen Metabolism in Patients with Peripheral Artery Disease

**DOI:** 10.3390/metabo11120814

**Published:** 2021-11-29

**Authors:** Zhe Li, Erin K. Englund, Michael C. Langham, Jinchao Feng, Kebin Jia, Thomas F. Floyd, Arjun G. Yodh, Wesley B. Baker

**Affiliations:** 1 Faculty of Information Technology, Beijing University of Technology, Beijing 100124, China; lizhe1023@bjut.edu.cn (Z.L.); fengjc@bjut.edu.cn (J.F.); kebinj@bjut.edu.cn (K.J.); 2 Beijing Laboratory of Advanced Information Networks, Beijing 100124, China; 3 Beijing Key Laboratory of Computational Intelligence and Intelligent System, Faculty of Information Technology, Beijing University of Technology, Beijing 100124, China; 4 Department of Radiology, University of Colorado Anschutz Medical Campus, Aurora, CO 80045, USA; erin.englund@cuanschutz.edu; 5 Department of Radiology, University of Pennsylvania, Philadelphia, PA 19104, USA; langhamc@pennmedicine.upenn.edu; 6 Department of Anesthesiology & Pain Management, The University of Texas Southwestern, Dallas, TX 75390, USA; thomas.floyd@utsouthwestern.edu; 7 Department of Physics & Astronomy, University of Pennsylvania, Philadelphia, PA 19104, USA; yodh@physics.upenn.edu; 8 Division of Neurology, Children’s Hospital of Philadelphia, Philadelphia, PA 19104, USA

**Keywords:** claudication, diffuse optical spectroscopy, exercise training, peripheral artery disease

## Abstract

Exercise training can mitigate symptoms of claudication (walking-induced muscle pain) in patients with peripheral artery disease (PAD). One adaptive response enabling this improvement is enhanced muscle oxygen metabolism. To explore this issue, we used arterial-occlusion diffuse optical spectroscopy (AO-DOS) to measure the effects of exercise training on the metabolic rate of oxygen (MRO_2_) in resting calf muscle. Additionally, venous-occlusion DOS (VO-DOS) and frequency-domain DOS (FD-DOS) were used to measure muscle blood flow (F) and tissue oxygen saturation (StO_2_), and resting calf muscle oxygen extraction fraction (OEF) was calculated from MRO_2_, F, and blood hemoglobin. Lastly, the venous/arterial ratio (γ) of blood monitored by FD-DOS was calculated from OEF and StO_2_. PAD patients who experience claudication (*n* = 28) were randomly assigned to exercise and control groups. Patients in the exercise group received 3 months of supervised exercise training. Optical measurements were obtained at baseline and at 3 months in both groups. Resting MRO_2_, OEF, and F, respectively, increased by 30% (12%, 44%) (*p* < 0.001), 17% (6%, 45%) (*p* = 0.003), and 7% (0%, 16%) (*p* = 0.11), after exercise training (median (interquartile range)). The pre-exercise γ was 0.76 (0.61, 0.89); it decreased by 12% (35%, 6%) after exercise training (*p* = 0.011). Improvement in exercise performance was associated with a correlative increase in resting OEF (R = 0.45, *p* = 0.02).

## 1. Introduction

Peripheral artery disease (PAD) is a common manifestation of the progressive stenosis of peripheral arteries. More than 200 million people have PAD worldwide, and the prevalence of PAD is ≥20% in individuals over the age of 80 years [1]. Approximately 30% of patients with PAD experience intermittent claudication, a walking-induced muscle pain primarily affecting the calves that is relieved only by rest [2]. Patients experiencing claudication generally have sedentary lifestyles and poor health-related quality of life [2,3]. Supervised exercise training programs have been shown to decrease the functional impairment caused by symptoms of claudication [4,5,6]. The underlying mechanisms for this improvement, however, are not well understood [7,8].

Claudication is triggered by limb ischemia that arises from large artery stenosis, vascular dysfunction, inflammation, and other factors [3,9]. Numerous investigators have studied the adaptive responses to exercise training in PAD patients that may delay the onset of limb ischemia [6,7,8,10,11,12,13]. One plausibly important adaptation mechanism is improved calf muscle oxygen metabolism [6,8,13,14,15]. With a higher metabolic rate of oxygen (MRO_2_), the level of anaerobic metabolism needed to supply the total energy load from walking is lower.

Thus, it is of interest to ascertain what underlying adaptations from exercise training enable improvements in MRO_2_, and in particular, whether these adaptations improve resting muscle MRO_2_. To date, direct blood flow measurements with optical diffuse correlation spectroscopy have provided evidence that exercise training increases the peak calf muscle blood flow reached during walking [16]; similar evidence is apparent from indirect time-to-minimum calf tissue oxygen saturation measurements with near-infrared spectroscopy [10,17,18,19,20]. Improvements in oxygen delivery from increased blood flow can sustain higher MRO_2_ levels during exercise. Another way to sustain higher MRO_2_ levels are adaptations that yield greater oxygen extraction per unit of blood delivered [6]. Herein, we investigate whether adaptive responses to exercise training impact resting calf muscle MRO_2_, blood flow (F), and oxygen extraction fraction (OEF).

Specifically, we incorporate the techniques of arterial-occlusion and venous-occlusion diffuse optical spectroscopy (i.e., AO-DOS and VO-DOS) into a randomized clinical study to ascertain the effects of a 3-month supervised exercise-training program on resting calf muscle MRO_2_ and F. Using Fick’s law [21,22], we calculated OEF from MRO_2_, F, and blood hemoglobin (C) measurements. The exercise training effects on resting calf muscle tissue oxygen saturation (StO_2_), total hemoglobin concentration (THC), and blood volume fraction (f_BV_ = THC/C) were also determined from FD-DOS and C measurements.

Importantly, our knowledge of OEF and StO_2_ enable the direct calculation of the ratio of venous and arterial blood monitored by FD-DOS (γ). In contrast to pulse oximetry, the StO_2_ measurement is a weighted average of arterial and venous oxygen saturations [21]: StO_2_ = (1 − γ) SaO_2_ + γ SvO_2_. The γ ratio thus informs the interpretation of absolute StO_2_ levels. Furthermore, a change in γ will result in a change in StO_2_, even if SaO_2_ and SvO_2_ are constant. One study has measured γ for FD-DOS cerebral oximetry in children with congenital heart disease [23]. To our knowledge, measurements of γ for the calf muscle have never been reported. Moreover, measurements of whether γ changes from exercise training have never been reported.

We hypothesize that exercise-training increases resting muscle oxygen metabolism and that these increases are driven by enhanced calf muscle oxygen extraction per unit of blood delivered. We also hypothesize that γ remains constant before and after exercise training.

## 2. Results

The exercise and control groups were well-matched, i.e., in terms of baseline demographics and performance variables (Table 1). The exercise group had a slightly higher near-surface tissue layer thickness above the calf muscle, though this difference was not significant.

Average pre-exercise training measurements of resting calf muscle metabolic rate of oxygen (MRO_2_), blood flow (F), oxygen extraction fraction (OEF); calculated from MRO_2_ and F data, tissue oxygen saturation (StO_2_), and total hemoglobin concentration (THC) across both the exercise and control group populations are reported in Table 2. Additionally, Table 2 reports the average measurements of blood hemoglobin concentration (C), calf muscle blood volume fraction (f_BV_), venous/arterial ratio of blood monitored by FD-DOS (γ; calculated from StO_2_ and OEF data), the recovery half-time of deoxy-hemoglobin (Hb) (i.e., time duration to half-minimum) for Hb to return to baseline after arterial occlusion, calf muscle tissue reduced scattering coefficients (µ_s_’; at 685, 785, 830 nm wavelengths), and the differential pathlengths (L) of light transport through the calf muscle (see Section 4.1). Note, there were no differences between the exercise and control groups in the pre-exercise measurements.

The peak walking time for patients in the exercise group (patients that received 3 months of supervised exercise training) significantly increased after exercise training (*p* < 0.001), i.e., the average 3-month/baseline ratio was 1.99 (1.24, 2.67). There was no accompanying change, however, in the ankle brachial index, i.e., the average 3-month/baseline ratio of 1.01 (0.88, 1.24) was not different from unity (*p* = 0.50). (The average post-exercise PWT and ABI for patients in the exercise group were 857 (730, 1178) s and 0.63 (0.50, 0.81).) For patients in the control group, the corresponding changes in peak walking time and ankle brachial index were −0.91 (−0.86, 1.12) and 1.00 (0.94, 1.10), respectively; these changes were not significant. (The average “post-intervention” PWT and ABI for patients in the control group were 343 (180, 567) s and 0.63 (0.56, 0.68).)

Exercise training did not alter blood hemoglobin and total hemoglobin concentration. The average 3-month/baseline C ratios of 1.01 (1.00, 1.02) and 1.02 (0.99, 1.03) for the exercise and control groups, respectively, were not different from unity, and for the FD-DOS THC metric, the average 3-month/baseline THC ratios of 1.08 (0.93, 1.13) and 1.00 (0.92, 1.17) for the exercise and control groups were not different from unity. The recovery half-time of Hb was also not altered by exercise training (i.e., the average 3-month/baseline ratios for the exercise and control groups were 0.91 (0.82, 1.32) and 0.92 (0.75, 1.23), which were both not different from unity). Additionally, the thickness of the near-surface tissue layer overlaying the muscle was not altered by exercise training (i.e., the average 3-month/baseline ratios for the exercise and control groups were 1.00 (0.97, 1.09) and 0.99 (0.97, 1.07), which were both not different from unity).

**Figure 1:**
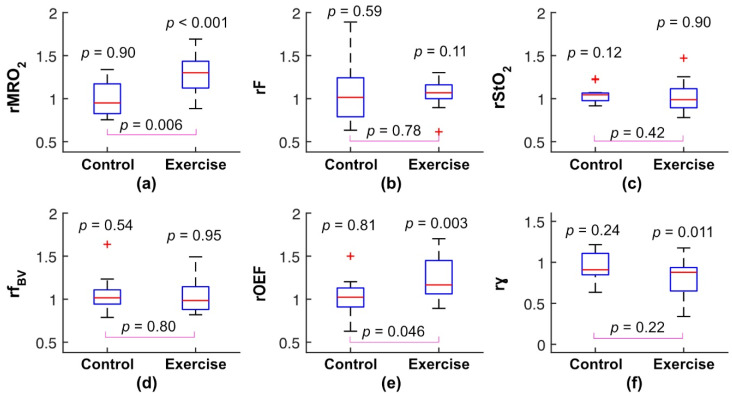
Optically measured changes in resting calf muscle metabolic rate of oxygen (rMRO_2_; (**a**)), blood flow (rF; (**b**)), tissue oxygen saturation (rStO_2_; (**c**)), blood volume fraction (rf_BV_; (**d**)), oxygen extraction fraction (rOEF; (**e**)), and venous/arterial ratio of blood monitored by FD-DOS (rγ; (**f**)) between the 3-month and baseline timepoints for the control (n = 14) and exercise (n = 14) peripheral artery disease (PAD) population groups. All changes are expressed as 3-month to baseline ratio, and all boxplots show the median and interquartile range (the + symbol in all boxplots indicates individual data points either greater than the third quartile plus 1.5× (interquartile range) or less than the first quartile minus 1.5× (interquartile range)). *p* values above the boxplots indicate whether the median change was different from 0, and *p* values between the boxplots indicate whether the median change in the exercise group is different from the median change in the control group.

The optical AO-DOS and VO-DOS results support our hypothesis that exercise training increases resting calf muscle metabolic rate of oxygen (Figure 1a) but does not significantly affect resting calf muscle blood flow (Figure 1b). The average rMRO_2_ (i.e., 3-month/baseline MRO_2_ ratio) of 1.30 (1.12, 1.44) across patients in the exercise group was higher than unity (*p* < 0.001), while the corresponding average rF of 1.07 (1.00, 1.16) was not statistically different from unity (*p* = 0.11). For patients in the control group, no differences were observed between the 3-month and baseline MRO_2_ and F measurements. Further, the median rMRO_2_ was higher in the exercise group than it was in the control group (*p* = 0.006), while there was no difference in median rF between the exercise and control groups (*p* = 0.78).

Additionally, exercise training did not alter resting calf muscle tissue oxygen saturation and blood volume fraction (Figure 1c,d), but it did increase resting calf muscle oxygen extraction fraction (Figure 1e). The average rOEF of 1.17 (1.06, 1.45) across patients in the exercise group was higher than unity (*p* = 0.003), while the average rOEF of 1.02 (0.91, 1.13) across patients in the control group was not different from unity (*p* = 0.81). rOEF in the exercise group was higher than rOEF in the control group (*p* = 0.046).

Finally, our results do not support the hypothesis that the venous/arterial ratio of blood monitored by FD-DOS is the same before and after exercise training. The ratio actually decreased after exercise training (Figure 1f); the average rγ of 0.88 (0.65, 0.94) across patients in the exercise group was lower than unity (*p* = 0.011)). The decrease in γ suggests that a partial redistribution of calf muscle blood volume from veins to arterioles could have occurred, and the effect may be an explanation for why OEF increased, but StO_2_ remained unchanged after exercise training (see Section 3). Of note, the average rγ of 0.91 (0.85, 1.11) across patients in the control group was not different from unity (*p* = 0.24), but there was also no difference in the average rγ between the exercise and control groups (*p* = 0.22).

**Figure 2:**
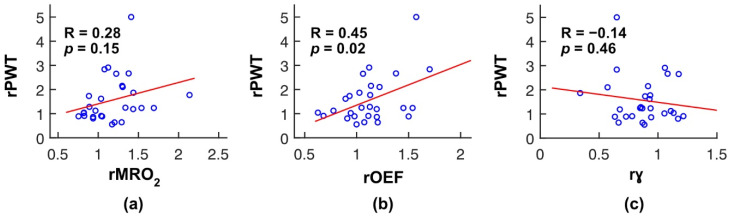
Across our cohort (n = 28 patients), changes in peak walking time (rPWT) were not correlated with changes in resting calf muscle metabolic rate of oxygen (rMRO_2_; (**a**)), were correlated with changes in resting calf muscle oxygen extraction fraction (rOEF; (**b**)) and were not correlated with changes in venous/arterial ratio of blood monitored by FD-DOS (rγ; (**c**)). All changes are expressed as 3-month to baseline ratio, and the solid lines are the linear best fits. *p* values indicate whether the slopes of the linear best fits are different from 0.

In a secondary analysis, we used linear regression to assess the correlations of changes in MRO_2_, OEF, and γ with improvement in exercise performance (Figure 2). The relative change in peak walking time (rPWT) was not significantly correlated with rMRO_2_ (R = 0.28, *p* = 0.15; slope of best fit line = 0.9 (95% CI, −0.3 to 2.1)), but was correlated with rOEF (R = 0.45, *p* = 0.02; slope of best fit line = 1.7 (95% CI, 0.4 to 3.0)). There was no correlation between rPWT and rγ (R = −0.14, *p* = 0.46; slope of best fit line = −0.7 (95% CI, −2.5 to 1.2)). Additionally, in another secondary analysis, there was no correlation between rMRO_2_ and rγ (R = −0.27, *p* = 0.17; slope of best fit line = −0.39 (95% CI, −0.96 to 0.18)).

## 3. Discussion

We found that exercise training increases the resting calf muscle MRO_2_ and OEF in PAD patients. This result, in combination with previous observations of larger fractional increases (relative to resting levels) in MRO_2_ and OEF during walking after exercise training [16], indicate that exercise-induced enhancement of absolute muscle MRO_2_ and OEF levels occur during walking. These enhancements likely delay the onset of limb ischemia caused by the energy load from walking, which may in turn delay the onset of claudication. We also measured the venous/arterial ratio of blood monitored by FD-DOS in calf muscle (i.e., γ), and we found that exercise training decreased γ.

Higher MRO_2_ levels are enabled by increases in muscle blood flow and/or by increases in muscle oxygen extraction per unit of blood delivered [22,24]. From previous investigations of PAD, there is evidence of exercise-induced increases in the peak calf muscle blood flow reached during walking [7,10,16,17,18,19,20]. In resting muscle, however, our results show that metabolism increases are predominantly driven by increases in oxygen extraction; significant changes in blood flow were not observed. We note that this blood flow finding is consistent with one previous clinical study of PAD patients that employed venous occlusion strain gauge measurements of flow [25].

The amount of oxygen extracted per unit blood delivered is the product of the arterial blood oxygen concentration (CaO_2_) and the calf muscle oxygen extraction fraction (OEF) [26]. Our results suggest that the oxygen extraction increase arises from increase in OEF, since blood hemoglobin concentration was not affected. Future work is needed, however, to investigate possible adaptations that could result in increased OEF, such as, for example, increased capillary blood volume, increased mitochondrial density, and/or enhanced blood O_2_ filterability due to decreased blood viscosity [6,7,8]. Indeed, the observed correlation between OEF changes and improvement in peak walking time, surprisingly, was higher than that for MRO_2_ changes. A future study with a larger sample size is warranted to confirm this finding. Possibly, adaptations that increase OEF may have other benefits not related to MRO_2_ that enhance exercise performance.

Surprisingly, measured StO_2_ was not altered by exercise training, despite its relation to OEF. Our observation of a decreased γ coefficient after exercise training (see Equation (4)) suggests a possible explanation for increases in OEF that are not accompanied by a reduction in StO_2_. StO_2_ can vary when OEF changes, but it will also vary if the relative arteriole and venous contributions to the StO_2_ signal change [21,23], i.e., when γ varies. Decreased γ can arise from reduced venous contribution or increased arterial contribution. For example, if no new vasculature is synthesized, then we speculate that muscle hypertrophy in subjects undergoing exercise training leads to greater venous compression, which in turn extrudes venous blood volume from the muscle. It is further possible that a redistribution of resting blood volume from veins to arterioles may help meet increased MRO_2_. We did not, however, observe a significant correlation between MRO_2_ and γ changes.

Since we found that the overall calf muscle blood volume fraction was not altered by exercise training, this drop in venous blood volume would be compensated by increased arteriolar and capillary blood volume (e.g., arising from endothelial dilation). A larger arterial contribution to the StO_2_ measurement would push StO_2_ higher, thereby counteracting the effects on StO_2_ from decreases in capillary and venous saturations caused by increased OEF. To our knowledge, this type of mechanism has not been reported in the literature. Note, however, the magnitude of the γ decrease that we measure is less than the magnitude of the OEF increase, which suggests that the γ change is only a partial explanation of the measured StO_2_ behavior.

Our measurements did not demonstrate a significant effect from exercise training on the recovery half-time of deoxy-hemoglobin after arterial occlusion. Higher maximal calf blood flow during reperfusion reduces the recovery time, but higher calf oxygen extraction during arterial occlusion increases the recovery time [27]. We speculate that our recovery time results are explained by these two competing factors roughly canceling each other. These results are consistent with our previously reported measurements of recovery time from maximal treadmill exercise [16]. Evidence from NIRS studies of the effects of exercise training on recovery time from maximal exercise, however, is mixed; some studies reported decreases in recovery time after exercise training [10,17].

Our average pre-exercise resting calf muscle oxygen metabolism measurements of 2.74 (2.24, 3.40) µmol/100 mL/min (or 0.06 (0.05, 0.07) mL/100 g/min) are roughly consistent with previous measurements of resting calf muscle oxygen metabolism in PAD patients (e.g., 0.05 (0.03, 0.07) mL/100 g/min [28], 0.10 (0.04, 0.16) mL/100 g/min [29]). They are also on a similar scale to previously reported resting calf muscle oxygen metabolism measurements in healthy adults, e.g., 0.03 (0.02, 0.05) mL/100 g/min [30], 0.05 (0.03, 0.06) mL/100 g/min [31], 0.04 (0.03, 0.06) mL/100 g/min [28].

Our average pre-exercise resting calf muscle blood flow measurements of 0.80 ± 0.28 mL/100 mL/min (mean ± SD) are consistent with previously reported VO-DOS calf muscle measurements in healthy adults, i.e., 0.88 ± 0.35 mL/100 mL/min [31], but lower than venous occlusion strain gauge calf muscle measurements in PAD patients, i.e., 3.54 ± 1.01 mL/100 mL/min [25]. Thus, future work directly comparing VO-DOS and strain gauge measurements in PAD patients is warranted.

Our average pre-exercise StO_2_ of 66.2 ± 7.5% (mean ± SD) is within the range of FD-DOS calf muscle measurements reported in the literature for PAD patients, e.g., 68 ± 6% [32] and 56 ± 16% [33], as well as for healthy adults, e.g., 72.7 ± 3.2% [31], 55.5 ± 9.9% [30], 62 ± 5% [34], 66.2 ± 11.4% [33], 72.0 ± 4% [32]. Our average THC of 106 ± 31 µM is within the range of previous FD-DOS calf muscle measurements performed in PAD patients, i.e., 68 ± 22 µM [32], and in healthy adults, i.e., 62 ± 21 µM [34], 48 ± 18 µM [30]. Finally, our average pre-exercise γ of 0.77 ± 0.18 in the calf muscle is similar to previous estimates of γ in the brain, i.e., 0.84 ± 0.21 [23]. It is also similar to the γ coefficients used by commercial NIRS oximeters, e.g., 0.7 or 0.75 [35].

The present study has limitations. First, the sample size was small, and we may be underpowered to detect increases in resting blood flow from exercise training. The median resting blood flow increase of 7% from exercise training (Figure 2b) may prove to be significant in a future study with a larger sample size. Our results do show, however, that increased oxygen extraction levels predominantly drive the observed increase in oxygen metabolism. Second, our calculations of OEF (Equation (3)) and γ (Equation (4)) assumed that arterial oxygen saturation is unity. We do not have the data to confirm the validity of this assumption. If arterial oxygen saturation is significantly lower than one, then the actual OEF will be higher and γ lower than our calculated values. It is also possible that a pre-exercise arterial oxygen saturation less than unity could become higher after exercise training. In this case, our reported rOEF change would overestimate the true change (i.e., the true change is the reported rOEF divided by rSaO_2_, where rSaO_2_ is the three-month/baseline ratio of the actual arterial oxygen saturations).

Third, our F and f_BV_ calculations assumed that the systemically measured blood hemoglobin concentration is the same as the blood hemoglobin concentration in the tissue volume sampled by the optical techniques. If blood hemoglobin is lower in the calf muscle microvasculature than in the systemic measurements, then the actual F and f_bv_ will be higher than the calculated values. We anticipate this source of error, however, should not alter our conclusions about relative F and f_BV_ changes from exercise, especially given our observation of constant systemic hemoglobin. Nevertheless, future work is needed to investigate whether blood hemoglobin in the calf muscle changes with exercise.

Fourth, variance in the adipose/skin thickness above the muscle can influence the absolute optical measurements [24,30,36]. Our primary conclusions, though, are based on relative changes, and the excellent intra-subject repeatability of calf muscle oxygen metabolism measurements observed in healthy adults [30] suggests that relative changes are robust to these effects. Finally, the mechanism by which exercise improves outcome is multifactorial [7], and future work is needed to elucidate whether enhanced muscle oxidative metabolism is a key factor behind exercise-induced improvement in claudication symptoms.

## 4. Materials and Methods

In this study, we carried out additional measurements on a subset of patients enrolled in a randomized control trial conducted at the University of Pennsylvania. The details of the trial’s study design are described elsewhere [16]. Subjects with intermittent claudication and a diagnosis of PAD were randomized to an exercise group or a control group using simple randomization. During screening, a history of intermittent claudication was confirmed with the San Diego Claudication and Walking Impairment Questionnaires [37]. Each subject further performed a graded treadmill walking protocol (i.e., the Gardner protocol; 2 mph, 0% initial grade with 2% grade increase every 2 min until maximal claudication [38]). The subject’s peak walking time (PWT) was defined as the walking time on the graded treadmill protocol at which ambulation could not continue because of maximal claudication. Within 1 month of the screening visit, subjects returned for a baseline assessment. To confirm reproducibility of baseline PWT, PWT was measured again, and subjects for which PWT between the two visits deviated by >25% were excluded. Each subject’s ankle-brachial index (ABI) and blood hemoglobin concentration (C) were also measured during the baseline assessment. The ABI is the ratio of ankle and brachial (arm) systolic blood pressures in the supine position, which was measured bilaterally with Doppler sonography [39]. Blood hemoglobin was measured from a venous blood gas sample acquired shortly after the treadmill walking protocol. Demographic information, including sex, age, body mass index (BMI), race, smoking status, history of diabetes, history of hypertension, and statin and cilostazol medication use, were recorded.

Subjects randomized to the exercise group performed three 60 min supervised exercise training sessions each week for a period of 3 months at the vascular laboratory of the University of Pennsylvania. As described elsewhere [40], during each training session, subjects walked on a treadmill at an initial speed of 2.0 mph to a mild to moderate pain level, stopped and rested until the claudication pain completely abated, and then resumed walking. This pattern was repeated for a total of 60 min. If subjects could walk longer than 8 min without rest, the treadmill walking became more challenging via grade and speed increases in subsequent training sessions [40]. At 3 months after the baseline assessment, ABI, PWT, and C were measured again in all subjects.

**Figure 3:**
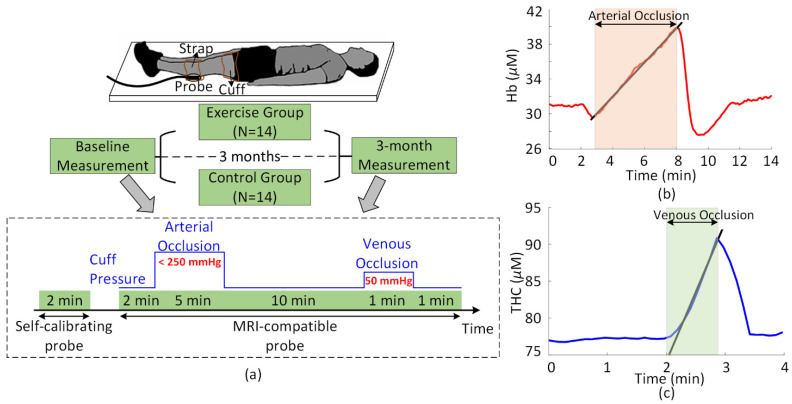
(**a**) Timeline of the randomized clinical study. Frequency-domain diffuse optical spectroscopy (FD-DOS), arterial-occlusion DOS (AO-DOS), and venous-occlusion DOS (VO-DOS) measurements of resting calf muscle in the most symptomatic leg were acquired in 28 peripheral artery disease subjects at two visits separated 3 months apart. After the initial visit, the subjects randomized to the exercise group did 3 months of supervised exercise training (see text). FD-DOS measurements were performed with a self-calibrating probe (see text). AO-DOS and VO-DOS measurements were performed with a MRI-compatible probe at the same location (MRI data was also collected during the AO- and VO-DOS measurements; this data is outside the scope of this paper). Arterial and venous occlusion were achieved with a pneumatic thigh cuff (blue solid line schematically shows the thigh cuff pressure). (**b**) Temporal plot of calf muscle deoxy-hemoglobin concentration (Hb) before/during/after arterial occlusion in a PAD patient. The slope of the Hb changes during the first minute of arterial occlusion indicates a calf muscle metabolic rate of oxygen of 6.68 µmol/100 mL/min. (**c**) Temporal plot of calf muscle total hemoglobin concentration (THC) before/during/after venous occlusion in a PAD patient. The slope of the THC change during venous occlusion, in combination with the measured blood hemoglobin concentration of C = 12.2 g/dL, indicates a calf muscle blood flow of 1.52 mL/100 mL/min.

In 28 subjects (14 in the exercise group, 14 in the control group) resting calf muscle metabolic rate of oxygen (MRO_2_) and blood flow (F) in the most symptomatic leg were noninvasively measured at baseline and at 3 months with the optical techniques of AO-DOS and VO-DOS (Figure 3). We additionally measured the tissue oxygen saturation (StO_2_), total hemoglobin concentration (THC), and multispectral tissue reduced scattering coefficients (i.e., µ_s_’ (λ) for λ = 685, 785, and 830 nm wavelengths) in resting muscle using frequency-domain DOS. As described in Section 4.1, these optical measurements were used in combination to calculate resting calf muscle oxygen extraction fraction (OEF), blood volume fraction (f_BV_), and venous/arterial ratio of blood monitored by FD-DOS (γ). The recovery half-time for deoxy-hemoglobin (i.e., time duration to half-minimum) to return to baseline after arterial occlusion was also measured (e.g., see Figure 3b). The thickness of the near-surface tissue layer overlaying the muscle (i.e., skin and adipose tissue) was measured for both the baseline and 3 month visits from MRI anatomical images at the site of application of the optical probe (probe site indicated by a MRI fiducial).

### 4.1. Diffuse Optical Measurements 

Optical measurements were performed using a custom-built three-wavelength (685, 785, 830 nm) frequency-domain diffuse optical spectroscopy instrument described elsewhere [24]. First, a self-calibrating optical probe, which we described previously in detail [16], was secured above the calf flexor, and the amplitude and phase of multispectral intensity modulated light were measured across 8 source-detector distances spanning 2.2–3.8 cm (these measurements were acquired every 8 s for 2 min). Using a semi-infinite homogeneous tissue model, this data was analyzed with a standard self-calibrating approach [24,41,42] to derive resting calf muscle StO_2_ and THC (a muscle water volume fraction of 70% was assumed [32]). Multispectral tissue reduced scattering coefficients of resting calf muscle were additionally derived (µ_s_’ (685 nm), µ_s_’ (785 nm), µ_s_’ (830 nm)).

For the arterial-occlusion and venous-occlusion DOS measurements, a second MRI compatible optical probe was secured to the same location above the calf flexor. Of note, these measurements were carried out in a 3T MRI scanner (the MRI data is outside the scope of this manuscript). A MRI fiducial was placed above the center of the probe to mark the probe’s position on the anatomical image for measurement of the skin and adipose tissue thickness. The amplitude and phase of multispectral intensity modulated light was monitored before/during/after a 5 min arterial occlusion and a 1 min venous occlusion at 2.5 cm source-detector distance (1 measurement was acquired every 8 s). The venous occlusion was initiated 10 min after the completion of arterial occlusion (Figure 3). Arterial and venous occlusions were achieved using a pneumatic tourniquet system (Hokanson, Inc, Bellevue, WA, USA) with a cuff placed around the midthigh. For arterial occlusion, the cuff was rapidly inflated to 75 mmHg above the measured systolic blood pressure, or 250 mmHg, whichever was lower. For venous occlusion, the cuff was rapidly inflated to 50 mmHg.

The semi-infinite modified Beer–Lambert approach was adopted to derive changes in calf muscle oxy-hemoglobin (HbO_2_) and deoxy-hemoglobin (Hb) from changes in the multispectral amplitude data [42,43]. These changes were relative to the 2 min baseline period immediately prior to arterial occlusion (see Figure 3). The multispectral differential pathlengths (i.e., L (685 nm), L (785 nm), L (830 nm)) used in the modified Beer–Lambert approach were calculated based on the frequency-domain DOS measurements of resting calf muscle absorption and reduced scattering coefficients, as described elsewhere [44].

Resting metabolic rate of oxygen (MRO_2_) was measured in the calf by calculating the rate of conversion of oxy-hemoglobin to deoxy-hemoglobin during arterial occlusion [29,45]:

MRO_2_ = 4 ∂Hb/∂t. (1)

Here, 4 molecules of O_2_ per molecule of HbO_2_ is assumed, and ∂Hb/∂t was determined from the slope of a linear regression fit of Hb versus time during the first minute of arterial occlusion (see Figure 3).

Resting blood flow (F) was measured from the rate of increase in total hemoglobin during venous occlusion [46,47]:

F = (∂THC/∂t)/C,(2)

where C is the hemoglobin concentration in the blood. C was measured from a venous blood sample drawn prior to the arterial and venous occlusions, and ∂THC/∂t was determined from the slope of a linear regression fit of THC versus time during the venous occlusion (see Figure 3).

Using Fick’s law [21,22], and assuming an arterial oxygen saturation of unity, we calculated the resting calf muscle oxygen extraction fraction from the MRO_2,_ F, and C measurements:

OEF = MRO_2_/(1.34 (C/100) F). (3)

Here, the units of MRO_2_, F, and C are mL O_2_/100 mL/min (note, x µmol O_2_/100 mL/min = 0.0224 x mL O_2_/100 mL/min), mL blood/100 mL/min, and g/(dL blood), respectively.

We then used the OEF calculated from Equation (3) and the measured StO_2_ to determine the venous/arterial of blood monitored by FD-DOS (γ). More precisely, StO_2_ measurements reflect a mixture of arterial, capillary, and venous blood, i.e., StO_2_ = k_a_ SaO_2_ + k_c_ ScO_2_ + k_v_ SvO_2_ [21]. SaO_2_, ScO_2_, and SvO_2_ are the arterial, capillary, and venous oxygen saturations, and k_a_, k_c_, and k_v_ are the respective weight of each compartment’s contribution to the total blood volume sampled by FD-DOS (k_a_ + k_c_ + k_v_ = 1). Assuming that plasma oxygen concentration is negligible compared to bound oxygen concentration, OEF = (SaO_2_ − SvO_2_)/SaO_2_, or in terms of StO_2_, OEF = (SaO_2_ − StO_2_)/(γSaO_2_), where γ ≡ k_v_ + k_c_ k_w_ is the FD-DOS venous/arterial ratio [21]. Here, the constant k_w_ arises from making a simplification that represents ScO_2_ as a weighted average of SaO_2_ and SvO_2_, i.e., ScO_2_ = (1 − k_w_) SaO_2_ + k_w_ SvO_2_. Assuming again that SaO_2_ = 1,

γ = (1 − StO_2_)/OEF, (4)

where OEF is given by Equation (3).

Finally, we calculated the blood volume fraction (f_BV_) in the muscle tissue by taking the ratio of THC and C, i.e., f_BV_ = THC/C (of note, the molecular weight of hemoglobin, i.e., 64,500 g/mol, was used to convert C from units of g/dL to µM for this calculation).

### 4.2. Statistical Analyses

Statistical calculations were performed with MATLAB R2018a (MathWorks). All statistical tests were two-sided, and *p* < 0.05 was considered to indicate significance. Summary statistics are presented using medians and interquartile ranges. For our primary analyses, we examined the changes in optically measured MRO_2_, F, OEF, StO_2_, f_BV_, and γ between the baseline and 3-month timepoints for the exercise group and for the control group. Specifically, the relative 3-month/baseline ratios were computed for all changes. Wilcoxon sign-rank tests were used to assess whether these ratios were significantly different from one for the exercise group and for the control group. Wilcoxon rank-sum tests were also used to assess whether the ratios in the exercise group were significantly different from the ratios in the control group. We performed identical analyses to examine changes in ABI, PWT, C, recovery half-time for deoxy-hemoglobin, and near-surface tissue layer thickness between the baseline and 3-month timepoints.

For our secondary analyses, we used linear regression analyses across our entire cohort (*n* = 28) to estimate the associations between the optical metrics that significantly changed from exercise training (i.e., determined from primary analyses) and improvement in exercise performance. Improvement in exercise performance was defined as the relative 3-month/baseline ratio for PWT (i.e., rPWT). The Pearson correlations (R) between the relative 3-month/baseline ratios of the optical metrics and rPWT were computed, as well as the slopes and 95% confidence intervals of the best-fit lines. Finally, to estimate whether a redistribution in resting calf muscle blood volume is associated with increased oxygen metabolism, we performed an identical linear regression analysis to compare MRO_2_ and γ changes.

## 5. Conclusions

In a randomized study of 28 PAD patients with intermittent claudication, we employed optical techniques to demonstrate increased resting calf muscle oxygen metabolism levels from exercise training, which appear to be predominantly driven by increases in resting calf muscle oxygen extraction per unit of blood delivered. Exercise training did not alter the calf muscle blood volume fraction, though our results suggest a partial redistribution of calf muscle blood volume from veins to arterioles. Improvement in peak walking time on the treadmill was associated with a correlative increase in resting calf muscle oxygen extraction fraction. Future work is needed to better understand the underlying exercise-induced adaptations that increase resting muscle oxygen extraction.

## Figures and Tables

**Table 1:** Patient demographics and performance variables at baseline (data are given as n (%) or median (25th percentile, 75th percentile)). *p* values were computed using the Fisher exact test and Wilcoxon rank-sum test, as appropriate.

	Exercise	Control	*p* Value
Number of patients	14	14	
Male sex, n (%)	11 (79)	6 (43)	0.12
Ankle Brachial Index	0.60 (0.44, 0.76)	0.59 (0.52, 0.72)	0.93
Peak Walking Time, s	558 (318, 731)	350 (254, 470)	0.26
Age, years	66 (62, 70)	65 (60, 69)	0.63
BMI, kg/m^2^	28.9 (25.8, 29.6)	26.8 (22.8, 29.2)	0.22
Race, n (%)			
White	8 (57)	5 (36)	0.45
Black	5 (36)	9 (64)	0.26
Hispanic	1 (7)	0 (0)	>0.99
Risk factor history, n (%)			
Diabetes mellitus	6 (43)	3 (21)	0.42
Hypertensive	12 (86)	10 (71)	0.65
Former smoker (quit > 3 mo)	10 (71)	10 (71)	>0.99
Former smoker (quit < 3 mo)	1 (7)	0 (0)	>0.99
Current smoker	0 (0)	3 (21)	0.22
Medication use, n (%)			
Statin	10 (71)	11 (79)	>0.99
Cilostazol	5 (36)	2 (14)	0.38
Thickness of near-surface layer (skin and adipose tissue), mm	4.4 (3.8, 5.2)	3.7 (2.9, 4.6)	0.07

**Table 2:** Pre-exercise training measurements averaged across the entire cohort (N = 28).

	Median (25th %, 75th %)
MRO_2_, µmol/100 mL/min	2.74 (2.24, 3.40)
F, mL/100 mL/min	0.77 (0.64, 0.90)
OEF, %	43 (37, 50)
StO_2_, %	66 (60, 72)
THC, µM	112 (80, 131)
C, g/dL	13.8 (12.5, 14.8)
f_BV_, %	6.7 (4.7, 7.6)
γ	0.76 (0.61, 0.89)
Recovery half-time of Hb, s	77 (55, 119)
µ_s_’ (685 nm), 1/cm	6.50 (5.78, 7.08)
µ_s_’ (785 nm), 1/cm	5.88 (5.30, 6.68)
µ_s_’ (830 nm), 1/cm	5.31 (5.01, 6.26)
L (685 nm), cm	9.64 (8.99, 11.30)
L (785 nm), cm	9.73 (8.65, 10.56)
L (830 nm), cm	8.96 (7.93, 9.71)

## Data Availability

The data presented in this study are available on request from the corresponding author. The data are not publicly available due to the participant consent obtained as part of the recruitment process.
